# Immunological Aspects of Graves' Ophthalmopathy

**DOI:** 10.1155/2019/7453260

**Published:** 2019-11-12

**Authors:** Dominika Łacheta, Piotr Miśkiewicz, Alicja Głuszko, Grażyna Nowicka, Maria Struga, Ireneusz Kantor, Krzysztof B. Poślednik, Shafaq Mirza, Mirosław J. Szczepański

**Affiliations:** ^1^Department of Biochemistry and Pharmacogenomics, Faculty of Pharmacy, Medical University of Warsaw, Warsaw, Poland; ^2^Laboratory of Biochemistry and Clinical Chemistry at the Centre for Preclinical Research, Medical University of Warsaw, Warsaw, Poland; ^3^Department of Internal Medicine and Endocrinology, Medical University of Warsaw, Warsaw, Poland; ^4^Chair and Department of Biochemistry, First Faculty of Medicine, Medical University of Warsaw, Warsaw, Poland; ^5^Department of Otolaryngology, Medical Center for Postgraduate Education, Warsaw, Poland

## Abstract

The body's autoimmune process is involved in the development of Graves' disease (GD), which is manifested by an overactive thyroid gland. In some patients, autoreactive inflammatory reactions contribute to the development of symptoms such as thyroid ophthalmopathy, and the subsequent signs and symptoms are derived from the expansion of orbital adipose tissue and edema of extraocular muscles within the orbit. The autoimmune process, production of antibodies against self-antigens such as TSH receptor (TSHR) and IGF-1 receptor (IGF-1R), inflammatory infiltration, and accumulation of glycosaminoglycans (GAG) lead to edematous-infiltrative changes in periocular tissues. As a consequence, edema exophthalmos develops. Orbital fibroblasts seem to play a crucial role in orbital inflammation, tissue expansion, remodeling, and fibrosis because of their proliferative activity as well as their capacity to differentiate into adipocytes and myofibroblasts and production of GAG. In this paper, based on the available medical literature, the immunological mechanism of GO pathogenesis has been summarized. Particular attention was paid to the role of orbital fibroblasts and putative autoantigens. A deeper understanding of the pathomechanism of the disease and the involvement of immunological processes may give rise to the introduction of new, effective, and safe methods of treatment or monitoring of the disease activity.

## 1. Introduction

**Figure 1:**
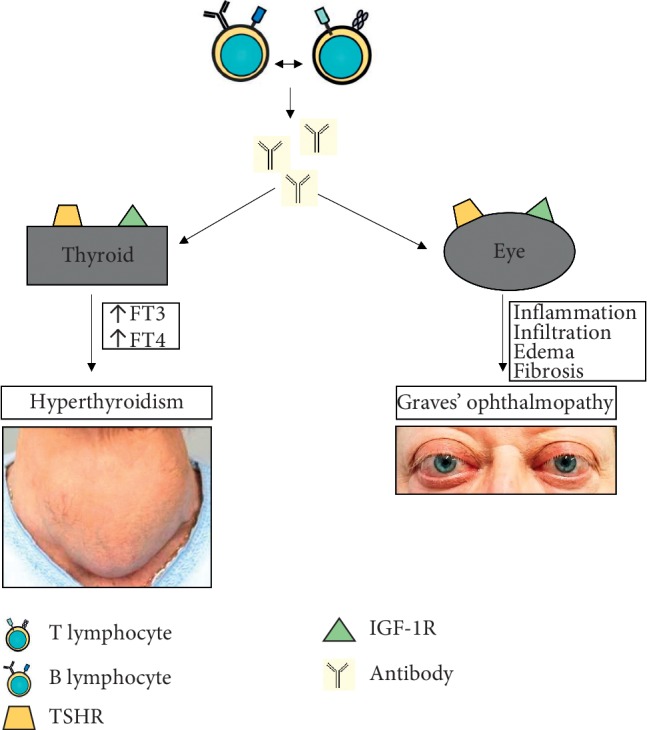
Pathogenesis of Graves' disease (GD) and Graves' ophthalmopathy (GO). GD is an autoimmune disease in which antibodies stimulate the thyroid to produce thyroid hormones leading to hyperthyroidism. One of the most common signs and symptoms is enlargement of the thyroid gland (goiter) while GO is the most frequent extrathyroidal involvement of GD. Inflammation and infiltration extraocular tissues result in edema and fibrosis of these tissues.

Graves' disease (GD) is the most common underlying cause of hyperthyroidism, and the incidence of new cases is estimated at 20 to 50 per 100,000 people per year [1]. It is a multifactorial disease, influenced by genetic, environmental, and endogenous factors. The peak in the disease occurrence is between the ages of 30 and 50 years, but it can occur at any age and affects women more often than men [2]. The cause of hyperthyroidism in GD is circulating autoantibodies directed against the thyrotropin receptor (TSHR), which mimic the action of TSH and excessively activate thyroid follicular cells and consequently stimulate the secretion of thyroid hormones (triiodothyronine and thyroxine), thereby inducing thyroid growth and its vascularization [3]. These processes trigger the development of hyperthyroidism symptoms such as anxiety, fatigue, nervousness, weight loss, moist skin, hair loss, muscle weakness, and palpitations. The extrathyroidal symptoms include localized dermopathy, acropachy, and ophthalmopathy, edematous-infiltrative changes involving orbital soft tissues described as thyroid-associated orbitopathy (TAO), and thyroid eye disease or Graves' ophthalmopathy (GO) since more than 90% are due to GD [4]. GO, defined as an autoimmune inflammatory disorder involving the orbit, is observed in about 2 subjects per 10,000 a year and in 25–50% of patients with GD [5, 6]. Although these patients are predominantly hyperthyroid (90%), patients with GO may also be euthyroid (5%) or hypothyroid (5%) [7]. It is observed that the pathological autoimmune reaction is directed against cross-reactive autoantigens in the thyroid and retrobulbar tissues [6, 8]. Significant involvement of cytokines and immunological mechanisms in the pathogenesis of GO is suggested. Tissue infiltration by cytokine-producing inflammatory cells and extensive remodeling of the eye soft tissues results in a phenotypic picture of the disease (Figure 1). Clinical signs and symptoms include double vision, retracting eyelids, edema, proptosis, and erythema of the conjunctival and periorbital tissues [6]. According to the recommendations of the European Group on Graves' Orbitopathy (EUGOGO), GO is distinguished into three levels of severity: mild, moderate to severe, and sight-threatening [9]. Treatment depends on the GO severity and includes immunosuppressive therapy, orbital irradiation, and surgery (endoscopic orbital decompression). Understanding the role of the immune system in GO may enable the introduction of new therapeutic options in the future.

## 2. Pathogenesis

Similarly to GD, at the base of GO is the autoimmune response in which the sensitive T cells, as well as autoantibodies against a common autoantigen of the thyroid and retrobulbar tissues, play an important role [10]. This common antigen may be the TSH receptor, as it has been also expressed on fibroblasts and orbital preadipocytes [11]. A correlation between the degree of ocular changes and the level of stimulatory antibodies directed against TSHR (TRAb) has been reported [12]. It has been suggested that another autoantigen may be the insulin-like growth factor-1 receptor (IGF-1R), as immunoglobulins of GD patients may activate the IGF-1R [13, 14]. Autoantibodies directed against this receptor contribute to the activation of orbital fibroblasts in GO, and the increased expression of the IGF-1R has been shown in patients with GD in both the thyroid tissue and the orbital tissues. Varewijck et al. demonstrated a diminished stimulating activity of IGF-1R through the depletion of immunoglobulins of GD patients [15]. Although these antibodies against IGF-1R are potentially implicated in GO development, there are some discrepancies regarding this speculation. Minich et al. have obtained data that do not confirm that the circulation of stimulating antibodies (against IGF-1R) in the patient's blood aggravates GD, nor their usefulness as a diagnostic parameter of the disease [16].

**Figure 2:**
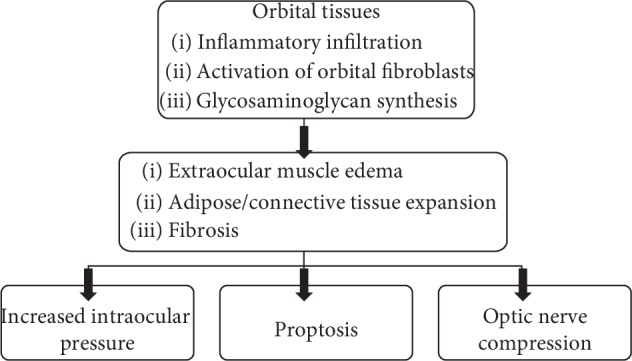
Pathogenesis of edematous-infiltrative changes. Inflammatory infiltration in periocular tissues and activity of orbital fibroblasts lead to expansion and remodeling of tissues. Increased intraocular pressure within the inflexible bony orbit results in proptosis and can contribute to developing optic nerve compression.

The main processes involved in the pathogenesis of thyroid-associated orbitopathy are cytokine production and inflammation, hyaluronan synthesis, adipogenesis, and myofibrillogenesis. The main sites of ongoing inflammation are the orbital adipose tissue and fibrous tissue of extraocular muscles [17]. The orbital tissues are infiltrated by activated mononuclear cells, such as T cells, and to a lesser extent by plasmocytes, macrophages, and mast cells. Cytokines produced by leukocytes, such as IFN-*γ*, IL-1*α* (IL-5), and leukoregulin (lymphokine, produced by activated lymphocytes), lead to the synthesis of glycosaminoglycans (GAG) [18]. The accumulation of GAG leads to extraocular muscle edema [19]. By means of inflammatory mediators (cytokines) or direct cellular interaction, orbital fibroblasts are activated, which exhibit different morphological and functional features as compared to fibroblasts in other localizations. Moreover, the activation of orbital fibroblasts by TRAb indicates the link between GD and GO [20, 21]. Activated orbital fibroblasts proliferate, differentiate into adipocytes and myofibroblasts, and play a key role in the production of the extracellular matrix. Excessive orbital fibroblast activity contributes to expansion, remodeling, and fibrosis of the orbital tissues. In the active phase of orbital changes, as a result of inflammatory cell infiltration and edema, the volume of tissues surrounding the eyes augments, in turn leading to an increase in the intraocular pressure [18]. As a consequence, the eyeball moves beyond the bony edges of the orbit. Moreover, optic nerve compression resulting in optic neuropathy, as well as impaired venous and lymphatic outflow from the orbit, can occur [22]. The final stage (inactive phase) of exophthalmos involves the fibrosis of the eye muscles (Figure 2).

## 3. Cytokine Production and Inflammation

**Figure 3:**
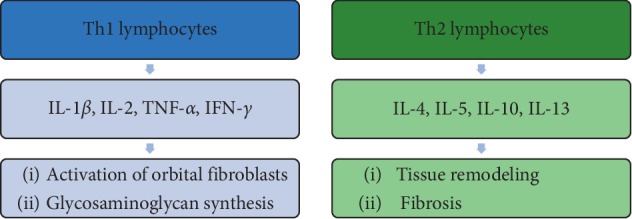
The proportion of T lymphocytes in the pathogenesis of Graves' ophthalmopathy. The initial phase of GO is characterized by increased activity of Th1 lymphocyte-producing cytokines that enhance fibroblast proliferation and GAG production. Th2 lymphocytes involved in the late phase participate in remodeling and fibrosis of periorbital tissues.

The inflammatory process in orbital tissues leads to migration and infiltration of immune cells, which resembles the process occurring within the thyroid gland. T cells enter the soft orbital tissue and release cytokines that contribute to reactivity and tissue remodeling [23]. The initial phase of GO is characterized by increased activity of Th1 lymphocytes, facilitating cell-mediated immunity and producing IL-1*β*, IL-2, TNF-*α*, and IFN-*γ* [24]. These proinflammatory cytokines enhance fibroblast proliferation and hydrophilic GAG production. Furthermore, the inflammatory process leads to the activation of Th2 lymphocytes, which release cytokines, such as IL-4, IL-5, IL-10, and IL-13, activating humoral reactions and the production of IgG [25]. The late phase of GO is characterized by tissue remodeling and fibrosis [26] (Figure 3).

**Figure 4:**
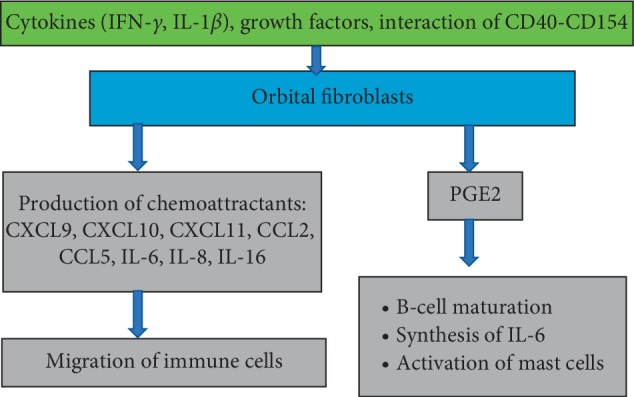
The participation of orbital fibroblasts in orbital inflammation. Cytokines, growth factors, and T cells stimulate orbital fibroblasts to produce chemokines and cytokines. PGE2 produced by orbital fibroblasts activates mast cells and B-cell maturation as well as stimulates the production of IL-6 by orbital fibroblasts.

Produced cytokines, chemokines, and growth factors have a huge impact on cells in orbital tissues. IFN-*γ* induces the production of CXCL9, CXCL10, and CXCL11 by fibroblasts, whereby the migration of lymphocytes to the orbital tissues is promoted [27]. In addition, IFN-*γ* stimulates the secretion of IL-1*β* and both (synergistically) stimulate the synthesis of GAG by orbital fibroblasts [28]. However, in contrast to IL-1*β*, IFN-*γ* inhibits adipogenesis of fibroblasts [29]. IL-1*β* has been shown to stimulate the orbital fibroblasts to produce IL-6, IL-8, CCL2, CCL5, and IL-16, which are chemoattractants for T and B cells, monocytes, and neutrophils [30, 31] (Figure 4).

Besides lymphocytes, macrophages, and thyrocytes, orbital fibroblasts also express the costimulatory protein CD40 [32]. The interaction between CD40 ligand (CD154) localized on T cells and the CD40 molecule on the orbital fibroblast surface stimulates the production of various inflammatory mediators (such as IL-1*α*, IL-6, IL-8, CCL2, and PGE2) by orbital fibroblasts as well as the activity and proliferation of these cells [32]. Prostaglandin E2 (PGE2) participates in B-cell maturation, stimulates the production of IL-6 by orbital fibroblasts, and activates mast cells [33, 34]. The production of PGE2 by orbital fibroblasts is also promoted by leukoregulin, IL-1*β* (released by macrophages and fibroblasts), and IFN-*γ* (secreted by activated T cells) [28, 35]. The process of recruitment of autoreactive T lymphocytes is supported by locally produced or circulating adhesion molecules, and the expression of these molecules is induced by cytokines [36]. IL-1*α*, IL-1*β*, TNF-*α*, IFN-*γ*, and also CD40-CD154 interaction enhance the expression of intercellular adhesion molecule (ICAM-1) on orbital fibroblasts [30, 37, 38]. Adhesive molecules activate T cells and enhance their recruitment, resulting in an increased cell response and development of the active phase of ophthalmopathy. Elevated levels of L-selectin and ICAM-1 have been reported in patients in the active phase of the disease [39].

It is suggested that the cause of the development of GO is a lack of regulatory T lymphocytes (Tregs) control over the inflammatory reaction directed against self-tissues (antigens) [40]. Tregs are responsible for suppressing the immune response by the release of IL-10 and TGF-*β* [41]. Under physiological conditions, Tregs destroy autoreactive T lymphocytes, directed against thyroid follicular cell antigens [42, 43]. Glick et al. demonstrated an impaired suppressor function of Treg lymphocytes in patients with autoimmune thyroid disease (GD or Hashimoto's disease), who did not receive glucocorticosteroids for a minimum of six months [44]. Klatka et al. reported that patients with GD were characterized by a lower number of Tregs and a higher Th17 lymphocyte count compared to healthy subjects [45]. The significant contribution of Th17 lymphocytes to inflammatory infiltration is also suggested as their role in autoimmune diseases has been demonstrated [46, 47]. The elevated concentration of Th17 lymphocytes in the peripheral blood of GO patients was reported, but there are no data on the presence of Th17 lymphocytes in the inflammatory infiltration of orbital fat.

## 4. Hyaluronan Synthesis

An important feature of the processes occurring in retro-ocular connective tissue, which affects the clinical picture of ophthalmopathy, is the synthesis of large amounts of GAG by orbital fibroblasts [48]. In particular, the accumulation of hyaluronan acid and collagen contributes to the retrobulbar tissue edema. *In vitro* culture of orbital fibroblasts treated with IFN-*γ* was characterized by higher production of GAG compared to the dermal fibroblasts culture [49]. Similar results were obtained using leukoregulin as a stimulant [50]. The effect of inflammatory mediators, such as IL-1, TNF-*α*, IFN*γ*, TGF-*β*, IGF-1, PDGF (platelet-derived growth factor), and prostaglandins, on the stimulation of orbital fibroblasts for the production of hyaluronan is also indicated [30, 48, 51–53]. Han et al. reported that IL-4 and IFN*γ* enhance the effect of IL-1*β* on GAG production by orbital fibroblasts as they augment the induction of hyaluronan synthase-2 (HAS2) expression by IL-1*β* [28]. Hyaluronan synthases (HASs) expressed on the cell membrane are responsible for the regulation of hyaluronan synthesis [54]. In GO, the major isoform of HAS involved in the synthesis of hyaluronan is HAS2. The balance between synthesis and degradation reflects hyaluronan accumulation. Zhang et al. reported the production of hyaluronidase by orbital fibroblasts [55].

## 5. Adipogenesis and Myofibrillogenesis

A portion of the orbital fibroblasts is called preadipocytes since they possess the capability to differentiate into mature adipocytes, which distinguishes them from fibroblasts from other locations in the body. This may be due to the high expression of the peroxisome proliferator-activated receptors (PPAR*γ*) [56]. PPAR*γ* belongs to the nuclear receptors of adipocytes, which act as transcription factors and regulate homeostasis of lipids and glucose. Adipogenesis in orbital fibroblasts is enhanced by the activation of PPAR*γ* with rosiglitazone [57]. PPAR*γ* agonists stimulate not only adipogenesis but also the expression of TSHR in cultured orbital preadipocytes. Moreover, they inhibit orbital inflammation and the production of hyaluronan [58]. Microarray studies have shown an upregulation of adipocyte-related genes (genes encoding PPAR*γ*, IL-6, adiponectin, and leptin) in the orbit in GO. The activity of cyclooxygenase-2 (COX2) in activated T cells results in the production of proadipogenic prostaglandins (PPAR*γ* ligands) [59]. COX2 is upregulated in the orbit in patients with GO, and as a result, prostaglandins provoke the process of adipogenesis in orbital fibroblasts [60].

**Figure 5:**
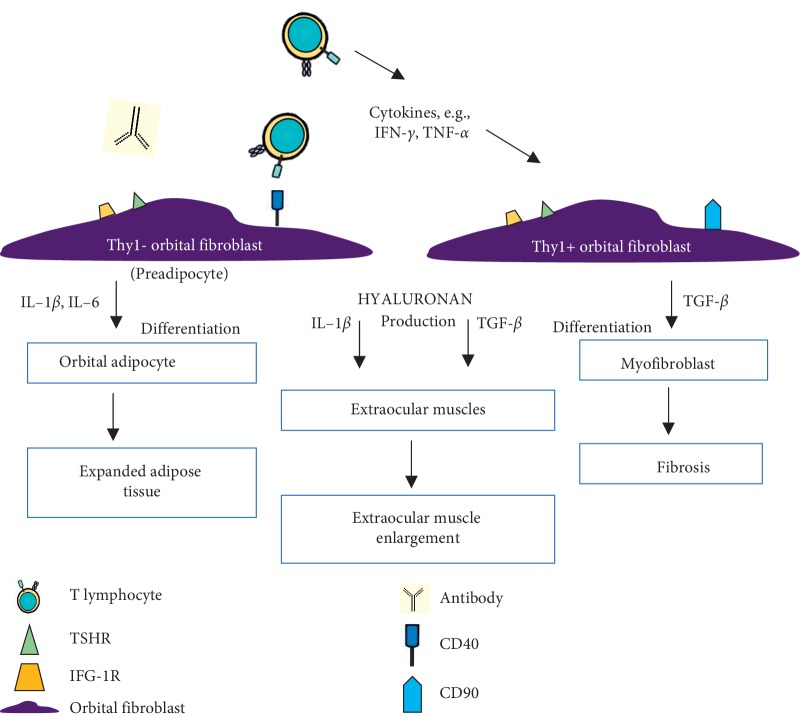
The participation of orbital fibroblasts in orbital tissue remodeling. Orbital fibroblasts express TSHR, IGF-1R, and CD40. Infiltrated immune cells, antibodies, secreted cytokines, chemokines, growth factors, and also CD40-CD154 interactions activate orbital fibroblasts. Inflammatory mediators (Il-1*β* and IL-6) that enhance adipogenesis activate Thy1− orbital fibroblasts to differentiate into adipocytes. And Thy1+ orbital fibroblasts (with CD90 expression), activated by TGF-*β*, differentiate into myofibroblasts. Proliferative activity of orbital fibroblasts, their differentiation, and capacity to synthesize extracellular matrix contribute to orbital tissue expansion, remodeling, and fibrosis.

Fibroblast subpopulations Thy1(CD90)+ and Thy1− can be distinguished based on the presence or absence of CD90 glycoprotein expression [61]. Thy1− fibroblasts have a strong ability to differentiate into adipocytes. Studies indicate that IL-1*β*, IL-6, and PGD2 stimulate fibroblasts towards adipogenesis [30, 52, 62]. It has been shown that this process is inhibited by TNF-*α* and IFN*γ*, but not by IL-4. These results agree with the claim that cytokines associated with Th1 lymphocytes are more involved in the early phase of ophthalmopathy rather than in the late phase associated with tissue remodeling and fibrosis. Thy1+ fibroblasts have the potential to differentiate into myofibroblasts, as demonstrated by fibroblasts stimulated by TGF-*β*, i.e., by a cytokine associated with Th2 lymphocytes [63, 64]. Myofibroblasts play a key role in muscle contraction and the accumulation of collagen in fibrotic tissue. Lehmann et al. have reported that adipocytic differentiation of Thy1− orbital fibroblasts can be inhibited by culture media from Thy1+ orbital fibroblasts, which produce antiadipogenic factors [63]. The involvement of adipose tissue or extraocular muscles in GO patients results from the proportion of Thy1+ and Thy1− orbital fibroblast populations and exposure to TGF-*β* or another stimulus [6] (Figure 5).

## 6. Putative Autoantigens and Potential Treatment

### 6.1. TSH Receptors

Hyperthyroidism associated with GD results from the action of autoantibodies directed against TSHR expressed on the surface of thyrocytes (thyroid epithelium). Studies have demonstrated the presence of the receptor in orbital adipose tissue and also suggested that the shared autoantigen hypothesis can explain the pathogenesis of GO (a common autoantigen of the thyroid and orbital tissues). Orbital adipose tissue of patients with GO (including euthyroid patients) is characterized by greater expression of TSHR than control tissues from people without GD [65, 66]. An elevated level of TSHR has been also noticed in pretibial connective tissue from patients with thyroid-associated dermopathy [67]. Some studies have shown that the level of antibodies against TSHR (TRAb) correlates with the clinical activity and severity of GO [68, 69]. Active GO is associated with a higher expression of TRAb compared to inactive GO. It is suggested that the extrathyroidal and thyroidal TSHR exhibit similar properties [70]. The response of orbital fibroblasts to TRAb is augmented by PDGF-AB and PDGF-BB, whereas TGF-*β* reduces TSHR expression [51, 71]. TSH, TRAb, and GD-IgG activate orbital fibroblasts and initiate cAMP and PI3K (phosphoinositide 3-kinase) signaling and the production of hyaluronan, ICAM-1, and cytokines, e.g., IL-6, IL-8, CCL2, and CCL5 [72]. In addition, the activation of TSHR induces adipogenesis in orbital fibroblasts [73].

Studies indicate that enhanced *de novo* adipogenesis in the orbit of GO patients increases TSHR expression in this tissue. Cultured orbital fibroblasts under adipogenic conditions have shown higher TSHR expression in mature fat cells than in preadipocyte fibroblasts [74]. Furthermore, PPAR*γ* agonist rosiglitazone and adipogenic conditions trigger the enhanced expression of TSHR and adipocyte-associated genes (adiponectin, leptin, and PPAR*γ*) [57, 65]. Similar findings have been obtained in orbital adipose tissue. In addition, monoclonal TRAbs stimulate adipogenesis in orbital preadipocyte fibroblasts, which indicates the involvement of autoantibodies not only in the overproduction of thyroid hormones in GD but also in an orbital adipose tissue volume increase in GO.

Smith and Hoa have discovered that purified immunoglobulins from patients with GD (GD-IgG including TRAb and other IgGs) participate in the production of hyaluronan [75]. They found that GD-IgG enhances hyaluronan synthesis in GO orbital fibroblasts (through IGF-1R) whereas such properties have not been demonstrated for human recombinant TSH (hrTSH). In addition, only orbital fibroblasts that have undergone adipocyte differentiation are induced to hyaluronan production by GD-IgG, but not by hrTSH [72, 76]. On the other hand, Zhang et al. have shown that, in undifferentiated orbital fibroblasts (not in GO fibroblasts), bovine TSH and TRAb stimulate hyaluronan synthesis [54]. They also demonstrated that GO orbital fibroblasts containing the transfected TSHR-activating mutation increase hyaluronan production.

Due to the fact that the TSHR plays an important role in the pathogenesis of GD, it is believed that this receptor may be a therapeutic target for the treatment of GD [77]. Considering the orbital fibroblast activation through TSHR signaling, small-molecule TSHR antagonists can be used to block signal transduction [78]. These molecules have been found to inhibit cAMP production in human thyrocytes induced by TSH and GD-IgG [79]. TSHR-blocking monoclonal antibodies inhibit hyaluronan production and adipogenesis in cultured human orbital fibroblasts [80]. TRAb K1-70 has antagonist activity and can be useful in the inhibition of stimulating TRAb in GD patients [81]. ATX-GD-59 is an apitope that decreases the production of stimulating TRAb and demonstrates potential for the prevention and treatment of GO [82]. Apitopes—antigen processing independent epitopes—mimic naturally processed CD4+ T-cell epitopes. Regulatory-like T cells (type 1) with immunosuppressive features are induced after the administration of apitopes.

### 6.2. Insulin-Like Growth Factor-1 Receptor (IGF-1R)

Another crucial autoantigen potentially involved in the pathogenesis of GO is IGF-1R. This receptor is expressed in many tissues, particularly in the thyrocytes and orbital adipose tissue in patients with GD and GO. It belongs to the tyrosine kinase receptors and is involved in processes such as cellular metabolism, growth, apoptosis, and immunity [77]. It also plays a role in the activation of T and B cells. Studies show higher IGF-1R expression in GO orbital fibroblasts than in normal cells [83]. Increased expression of IGF-1R has been found not only in the retro-orbital tissue of GO patients but also in the thyroid tissue of GD patients [14]. The stimulation of GO orbital fibroblasts by GD-IgG leading to the synthesis of T-cell chemoattractants, i.e., IL-16 and chemokine RANTES is attenuated by autoantibodies blocking IGF-1R or by transfecting fibroblasts with a dominant negative mutant IGF-IR. This draws attention to the vital role of signaling through IGF-1R in this process [84]. The chemoattractant effect contributes to the recruitment of inflammatory cells into the orbital tissues and promotes the autoimmune response. IGF-1R is found to participate in the differentiation of orbital fibroblasts into adipocytes and in the synthesis of hyaluronan through the action of autoantibodies directed against this receptor [70].

Research indicates that IGF-1 and TSH cooperate in the differentiation and metabolism of thyroid cells [85]. Their common location has been demonstrated in the membrane, in cytoplasmic and nuclear thyroid regions, and also in orbital fibroblasts. Tsui et al. have demonstrated that a monoclonal IGF-1R-blocking antibody inhibits kinase signaling induced by TSH. This antibody can also inhibit M22 (monoclonal TRAb) induced hyaluronan production by orbital fibroblasts. It can result from an association (physical and functional) between IGF-1R and TSHR [86]. Studies have shown that blocking IGF-1R through teprotumumab, a monoclonal antibody, inhibits IGF-1 and TSH action in fibrocytes and reduces the expression of IGF-1R and TSHR [87]. Teprotumumab infusions have great potential in reducing proptosis and the clinical activity score (CAS) in GO [88]. In 2016, the Food and Drug Administration described teprotumumab as a “breakthrough therapy.” At present, it is being evaluated in phase III RCT.

### 6.3. Other Potential Targeted Treatments

Antibodies targeting T cells can be used as a potential therapy since the participation of these cells in the pathogenesis of GO is crucial. Antibodies against CD3 (teplizumab and otelixizumab) lead to the depletion of T cells as in the case of type 1 diabetes [89]. Studies have also found that abatacept, a CTLA4 analogue, diminishes the activation of T cells. This approach was reported to be useful in corticosteroid-resistant rheumatoid arthritis [90]. Furthermore, the application of synthetic peptides in the silencing of autoimmune responses and the induction of T-cell tolerance to autoantigens has been used in experimental autoimmune encephalomyelitis in an animal model of multiple sclerosis [91]. However, none of these approaches connected with inhibiting T cells were investigated in autoimmune thyroid disease [92]. Because CD40-CD154 pathway participates in GD pathogenesis, the anti-CD40 antibody may be a promising approach in the treatment of GD. Iscalimab is one such immunomodulating, human, blocking anti-CD40 monoclonal antibody which can successfully treat Graves' hyperthyroidism [93].

Rituximab, a monoclonal antibody directed against CD20 on B cells, is actively investigated as it expresses an immunosuppressive effect. This monoclonal antibody decreases the production of TRAb [94]. Salvi et al. have demonstrated an improvement in GO activity and severity after the application of rituximab [95]. Although studies conducted by these researchers have also shown favorable effects of treatment by rituximab compared with intravenous methylprednisolone, Stan et al. did not confirm this in the prospective trial [5, 96]. However, long disease duration before treatment initiation may have significantly impacted different results of the mentioned researchers. It seems that rituximab may be vital in the case of a poor response to corticosteroids in patients with GO.

Another possible pathway in the treatment of GO is targeting TNF because of the impact of TNF on the production of MCP-1 by preadipocytes, which is crucial in attracting macrophages [97]. Adalimumab, a monoclonal antibody directed against TNF, was found to reduce inflammation in active GO and etanercept (soluble TNF receptor) can improve soft tissue changes [98, 99]. As TGF-*β* demonstrates a profibrotic effect, especially in patients with inactive GO, neutralizing this effect can be beneficial.

Serum concentrations of soluble IL-6 receptor are elevated in patients with active GO and correlate with disease activity [100]. Treatment with an IL-6 monoclonal antibody (tocilizumab) leads to decreased proptosis and improvement in eye muscle motility as well as in severity and activity in corticosteroid-resistant GO [101]. IL-1 is also markedly involved in the pathogenesis of GO. Studies carried out on cultured human orbital fibroblasts have shown that an antagonist of the IL-1 receptor (anakinra) inhibits hyaluronan production and decreases inflammation [102]. Potential therapeutic targets in GO are summarized in Table 1 [6, 103].

## 7. Conclusions and Future Prospects

**Figure 6:**
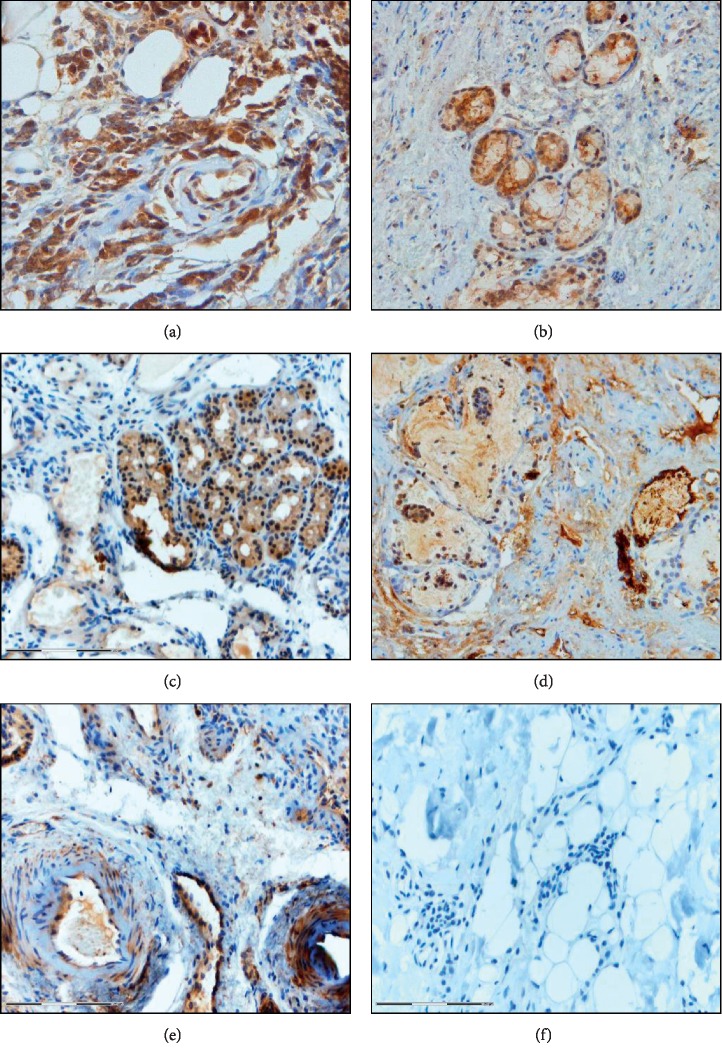
Immunohistochemistry on formalin-fixed paraffin-embedded tissue section of fat tissue of the eye socket obtained from patients who underwent endoscopic orbital decompression due to dysthyroid optic neuropathy: (a) TGF-*β* (×200); (b) TLR-4 (×100); (c) NF-kappa B (×100); (d) HIF-1*α* (×100); (e) IL-17 (×100); (f) isotype control (×100). The color reaction was visualized using DAB as a chromogen.

GD is an autoimmune disease underlying immune tolerance disorders and reactivity to thyroid autoantigens. One of the nonthyroid symptoms is GO, in which the autoreactive inflammatory process in the orbital tissues plays the main role. Extraocular muscles and connective tissues are infiltrated by immune cells. This inflammatory infiltration and cytokine production result in the activation of orbital fibroblasts, differentiation, and synthesis of GAG. As a consequence, muscle swelling, adipose tissue expansion, and fibrosis develop. Orbital fibroblasts exhibit particular features as they are a target for TSHR and IGF-1R autoantibodies and also possess the ability to differentiate into adipocytes and myofibroblasts. Our preliminary study indicates that, in the orbital adipose tissue of patients with GO, TGF-*β*, Toll-like receptor 4 (TLR-4), hypoxia-inducible factor-1*α* (HIF-1*α*), nuclear factor kappa B (NF-kappa B), and IL-17 are expressed (unpublished data). It is well known that the expression of these proteins is associated with increased fibrosis, inflammation, hypoxia, and autoimmunity (Figure 6). Toll-like receptors (TLR) are classified as pattern recognition receptors and exhibit expression on monocytes, macrophages, dendritic cells, B cells, and T cells. The signaling pathway activates NF-kappa B, leading to cytokine production. TLRs participate in the development of autoimmune and inflammatory diseases [104]. Liao et al. have reported that TLR-9 gene polymorphisms were associated with an increased risk of GO in male GD patients [105]. HIF-1*α* is activated in response to cellular hypoxia, which results in tissue remodeling in GO through activation of HIF-1*α*-dependent pathways in orbital fibroblasts. HIF-1*α* levels in these cells correlate with the clinical activity score of GO patients [106]. Due to insufficient knowledge regarding the pathomechanism of GO, there is no effective and safe method of treating this disease. The current treatment with the use of methylprednisolone pulses is effective in active moderate to severe GO in about 50% of cases and it carries the risk of complications, including fatalities (thromboembolic complications, sudden cardiac deaths, and severe liver damage) [107, 108]. An in-depth understanding of the function of immune cells as well as fibroblasts, adipocytes, and cytokines in GD patients may, in the future, help to define new treatment modalities or improve monitoring of the disease activity.

## Figures and Tables

**Table 1:** Potential therapeutic targets in GO [6, 103].

Target	Treatment	Potential benefit	
TSHR	TSHR-blocking antibody; TSHR antagonist	Inhibition of hyaluronan production and adipogenesis	[109]
IGF-1R	Teprotumumab—IGF-1R-blocking antibody	Inhibition of hyaluronan production and adipogenesis	[87, 88]
CD3	Teplizumab and otelixizumab—CD3 monoclonal antibodies	Induction of tolerance	[89]
CTLA4	Abatacept—CTLA4 analogue	Increased T-cell activation	[90]
CD20	Rituximab—CD20 monoclonal antibody	Increased TRAb production	[5, 94, 95]
TNF and TNF receptor	Adalimumab—TNF-blocking monoclonal antibody; Etanercept—soluble TNF receptor	Inhibition of hyaluronan production and inflammation	[99, 110]
TGF-*β*	TGF-*β*-blocking monoclonal antibody	Reduction in fibrosis	[111]
IL-6 receptor	Tocilizumab—IL-6 receptor monoclonal antibody	Inhibition of hyaluronan production and inflammation	[101, 112]
IL-1 receptor	Anakinra—IL-1 receptor antagonist	Inhibition of hyaluronan production and inflammation	[113]
